# Proteolysis and cartilage development are activated in the synovium after surgical induction of post traumatic osteoarthritis

**DOI:** 10.1371/journal.pone.0229449

**Published:** 2020-02-27

**Authors:** Ugur M. Ayturk, Jakob T. Sieker, Carla M. Haslauer, Benedikt L. Proffen, Manuela H. Weissenberger, Matthew L. Warman, Braden C. Fleming, Martha M. Murray

**Affiliations:** 1 Department of Orthopaedic Surgery, Boston Children’s Hospital, Harvard Medical School, Boston, Massachusetts, United States of America; 2 Department of Genetics, Harvard Medical School, Boston, Massachusetts, United States of America; 3 Department of Pathology, Caritas-Hospital Bad Mergentheim, Bad Mergentheim, Germany; 4 Department of Orthopaedics, Rhode Island Hospital, Warren Alpert Medical School of Brown University, Providence, Rhode Island, United States of America; Mayo Clinic Minnesota, UNITED STATES

## Abstract

Anterior cruciate ligament (ACL) transection surgery in the minipig induces post-traumatic osteoarthritis (PTOA) in a pattern similar to that seen in human patients after ACL injury. Prior studies have reported the presence of cartilage matrix-degrading proteases, such as Matrix metalloproteinase-1 (MMP-1) and A disintegrin and metalloproteinase with thrombospondin motifs 4 (ADAMTS-4), in the synovial fluid of injured or arthritic joints; however, the tissue origin of these proteases is unknown. The objective of this study was to identify transcriptional processes activated in the synovium after surgical induction of PTOA with ACL transection, and to determine if processes associated with proteolysis were enriched in the synovium after ACL transection. Unilateral ACL transection was performed in adolescent Yucatan minipigs and synovium samples were collected at 1, 5, 9, and 14 days post-injury. Transcriptome-wide gene expression levels were determined using bulk RNA-Sequencing in the surgical animals and control animals with healthy knees. The greatest number of transcripts with significant changes was observed 1 day after injury. These changes were primarily associated with cellular proliferation, consistent with measurements of increased cellularity of the synovium at the two-week time point. At five to 14 days, the expression of transcripts relating to proteolysis and cartilage development was significantly enriched. While protease inhibitor-encoding transcripts (*TIMP2*, *TIMP3)* represented the largest fraction of protease-associated transcripts in the uninjured synovium, protease-encoding transcripts (including *MMP1*, *MMP2*, *ADAMTS4*) predominated after surgery. Cartilage development-associated transcripts that are typically not expressed by synovial cells, such as *ACAN* and *COMP*, were enriched in the synovium following ACL-transection. The upregulation in both catabolic processes (proteolysis) and anabolic processes (cartilage development) suggests that the synovium plays a complex, balancing role in the early response to PTOA induction.

## Introduction

Knee injuries, particularly disruption of the anterior cruciate ligament (ACL), drastically increase the risk for post-traumatic osteoarthritis (PTOA) in human patients [1–3] and animal models [4, 5]. Clinical studies have supported catabolic changes in the articular cartilage occurring within the first few weeks of injury by identifying the breakdown products of articular cartilage in the synovial fluid, such as C-terminus of ¾ fragment short of type II collagen (C1, C2) after ACL injury or C-terminus of ¾ fragment long of type II collagen (C2C) after ACL reconstruction surgery[6, 7], as well as high levels of pro-inflammatory cytokines and extracellular matrix fragments in the synovial fluid of injured human knee joints[7–12]. Previous studies have also revealed increased Matrix metalloproteinase-1 (MMP-1), and A disintegrin and metalloproteinase with thrombospondin motif 4 (ADAMTS-4) proteins in the synovial fluid of injured or arthritic joints[13–15].

While the association of the subchondral bone and bone marrow lesions with osteoarthritis has been widely recognized [16], there is also increasing evidence for a causal role of the synovium in osteoarthritis[17–19]. The synovium is commonly regarded as a source of pro-inflammatory cytokines [19–21], which could affect articular cartilage health through the synovial fluid, ultimately facilitating the proteolysis of the articular cartilage extracellular matrix (ECM). ACL injuries in humans (prior to surgical treatment) have been shown to be associated with injury to the capsule and its underlying synovium in most ACL-injured patients [22, 23]. The porcine surgical ACL transection model, which incorporates a controlled synovial injury with the ACL injury, results in a similar pattern of post-traumatic osteoarthritis as is seen in the human condition[3, 5]. Prior reports in a minipig model suggest upregulation of proteolytic and cartilage development process networks in the synovium at 1 and 4 weeks after surgical transection of the ACL[19]. In addition, early anti-inflammatory treatment with intraarticular triamcinolone acetonide injections prevented the increase in collagen fragments (C-telopeptide of type II collagen (CTX-II)) in the synovial fluid of human patients with acute ACL injury[24], and C1 and 2C fragments in minipigs following ACL transection surgery[25], further supporting the link between synovial inflammation and cartilage ECM proteolysis. However, the early timing of synovial changes, and thus the optimum timing for possible interventions, remains unknown.

Despite advances in our understanding of the molecular mechanism of PTOA[26], the full response of the synovium to joint injury is incompletely understood. Transcriptome-wide gene expression analyses, using technologies such as microarrays or RNA-Sequencing (RNA-Seq), can assess the expression levels of all known genes within a cell or tissue sample. As truly healthy human control tissues are typically unavailable, analysis of gene expression in the synovium has focused on the later stages of osteoarthritis, where tissue samples can be readily obtained during total knee replacement surgery[27]. However, these late stage samples are unable to provide insight into the early, possibly disease-initiating mechanisms that occur in the first two weeks after a PTOA-initiating event.

Animal models offer the means of circumventing some of the limitations associated with human studies, since sample collection can be systematically controlled, and healthy control tissues more easily obtained. Several *in vivo* animal models of PTOA utilize ACL transection as the initiating event[5, 28, 29]. Large animals are well suited for these studies, since their tibiofemoral and patellofemoral joints are more similar to human knees in terms of size, cartilage thickness and subchondral bone organization[30]. We validated a pig ACL transection model that develops microscopic features of osteoarthritis (including cell cloning, GAG loss and surface fissuring) at four weeks after surgery[31], and that progresses over time to macroscopic PTOA in the same pattern as human patients after an ACL injury (including full thickness cartilage loss involving predominantly the medial compartment of the knee)[5]. Thus, this model provides a reliable method to study the mechanisms by which an acute joint injury progresses to PTOA.

The objective of this study was to identify the transcriptional events elicited in the synovium during the acute phase (first 14 days) following knee injury. We hypothesized 1) that significant enrichment of protease-related networks would be found in the synovium after ACL transection, and 2) that at least one of the significantly upregulated genes in the synovium would encode a protease found in the synovial fluid after injury with known action against ECM proteins, namely either *MMP1* or *ADAMTS4*[13–15].

## Materials and methods

### Surgical procedures

Twenty-three adolescent male Yucatan minipigs were sourced from an AAALAC accredited company (Sinclair Research, Columbia MO), 12–15 months of age, were subjected to a surgical ACL transection procedure on one knee, and randomly allocated to euthanasia at one of four time points: day 1 (n = 6), day 5 (n = 5), day 9 (n = 6), and day 14 (n = 6) post-surgery. The surgical ACL transection in the porcine model has been previously demonstrated to lead to both microscopic [31] and macroscopic [5] damage at one and twelve months, respectively, in a pattern that mimics what is seen in ACL-deficient patients[5]. The ACL transection procedure was performed as previously described[32, 33] under protocols approved by the Boston Children’s Hospital Institutional Animal Care and Use Committee (13-11-2543R). All animal procedures were performed following the ARRIVE guidelines under the direction of the animal care veterinarian. Surgery was performed under general anesthesia, and postoperative analgesics, including a Fentanyl 1–4 ug/kg transdermal patch, were administered to all animals for 72 hours post-operatively. Animals were monitored twice daily in the first post-operative week for signs of discomfort (limited or delayed weight bearing), activity and appetite, incision line assessment, hydration (by evaluating skin turgor). Additional analgesics were provided for any animals with signs of pain. Protocols were in place for early euthanasia for any animals that were unable to ambulate comfortably or had evidence of active infection at the operative site. No mortality occurred outside of the planned euthanasia at the end of the study.

The ACL was exposed by performing a medial arthrotomy and partial resection of the fat pad, followed by transection of the ACL at the junction of the proximal and middle thirds using a scalpel blade. The injured joint space was irrigated with 500 cc of sterile saline during the procedure. Following surgery, the animals were permitted normal nutrition and *ad libitum* activity throughout the experimental period. When the animals were euthanized, the lateral synovium located a minimum of 2 mm away from any cartilage or meniscal surfaces was collected from the surgical limb, immediately frozen in liquid nitrogen or embedded in optimal cutting temperature (OCT) compound (for histology; Sakura Finetek, Torrance, CA), and stored at -80°C. Synovial tissue from the same location was harvested from both legs of 6 uninjured animals (n = 12) to serve as a control group.

### RNA-seq library preparation

Total RNA was extracted from the synovium through homogenization, phenol-chloroform separation and on-column purification using PureLink^™^ RNA Mini Kit (Life technologies, Carlsbad, CA). RNA samples were treated with DNAse I (PureLink^™^ DNase Set, Life technologies, Carlsbad, CA) and assessed with the 2100 Bioanalyzer (Agilent Technologies, Santa Clara, CA). RNA integrity numbers (RIN) were >6.7 for all included samples. The samples were enriched for polyA+ mRNA, reverse transcribed with random hexamers, ligated with indexed adapters and amplified with 15 cycles of PCR using the TruSeq RNA Sample Preparation Kit v2 (Illumina, San Diego, CA). Libraries were pooled (n = 8 libraries/lane) and sequenced on an Illumina HiSeq 2000 machine with 50 basepair paired-end reads to generate ~12–20 million reads/library.

### RNA-seq data analysis

Raw reads were mapped to the pig genome (Susscr3, released Aug. 2011) with RNA-Seq Unified Mapper (RUM)[34]. Reads uniquely aligned to the exons of each gene were counted with a custom R script that utilizes Rsamtools[35] and GenomicsFeatures packages, and used for differential expression analysis with edgeR[36]. Additionally, reads per kilobase of exon model per million mapped reads (RPKM) were calculated to evaluate relative expression of transcripts within the same library, and impose a lower threshold for detectability. The calculated *p*-values in the differential expression analyses were corrected for multiple hypothesis testing[37]. Changes in the expression of a transcripts were considered significant when *p*<0.05 (verified with sample-specific leave-one-out cross validation[38]) and the mean RPKM was >5 for one of the sample groups being compared.

RNA-Seq data were additionally analyzed using the MetaCore^™^ software suite and the proprietary *process network* ontology (Thomson Reuters, New York, NY). Significant changes (*p*<0.05 after adjustment for multiple hypothesis testing) in the activity of specific gene groups were reported as enrichment, regardless of the direction of change (i.e. increased or decreased mRNA abundance).

### Histopathological analyses of synovium

OCT-embedded synovium samples were cut in 6 μm sections containing both the intimal and the subintimal layers, and each section was stained with hematoxylin & eosin (MassHistology, Worcester, MA). Whole slide imaging was carried out on a VS120-S5 virtual slide system (Olympus, Tokyo, Japan) using a 20x objective in combination with a BX61VS microscope (Olympus). The ratio of the nuclear area to the total tissue area of the biopsy was calculated as a measure of cellularity (ImageJ 64-bit version 1.48, National Institutes of Health, Bethesda, MD). Good correlation has been previously found between the ratio of nuclear area to tissue area and the number of manually counted cells (R2 >0.49; data not shown). Microscopic scoring of the synovial biopsies was performed using the semiquantitative scoring system developed by Krenn and colleagues[39]. The group assignment for each slide was blinded during scoring. The assessment included the degree of intimal hyperplasia, stromal cellularity and inflammatory infiltration (each parameter from 0-absent to 3-strong)[39]. The microscopic sum score represented the sum of all parameters and ranged from 0–9 (0-no synovitis, 9-highest degree of synovitis).

## Results

### Differential expression in synovium following joint injury

**Fig 1:**
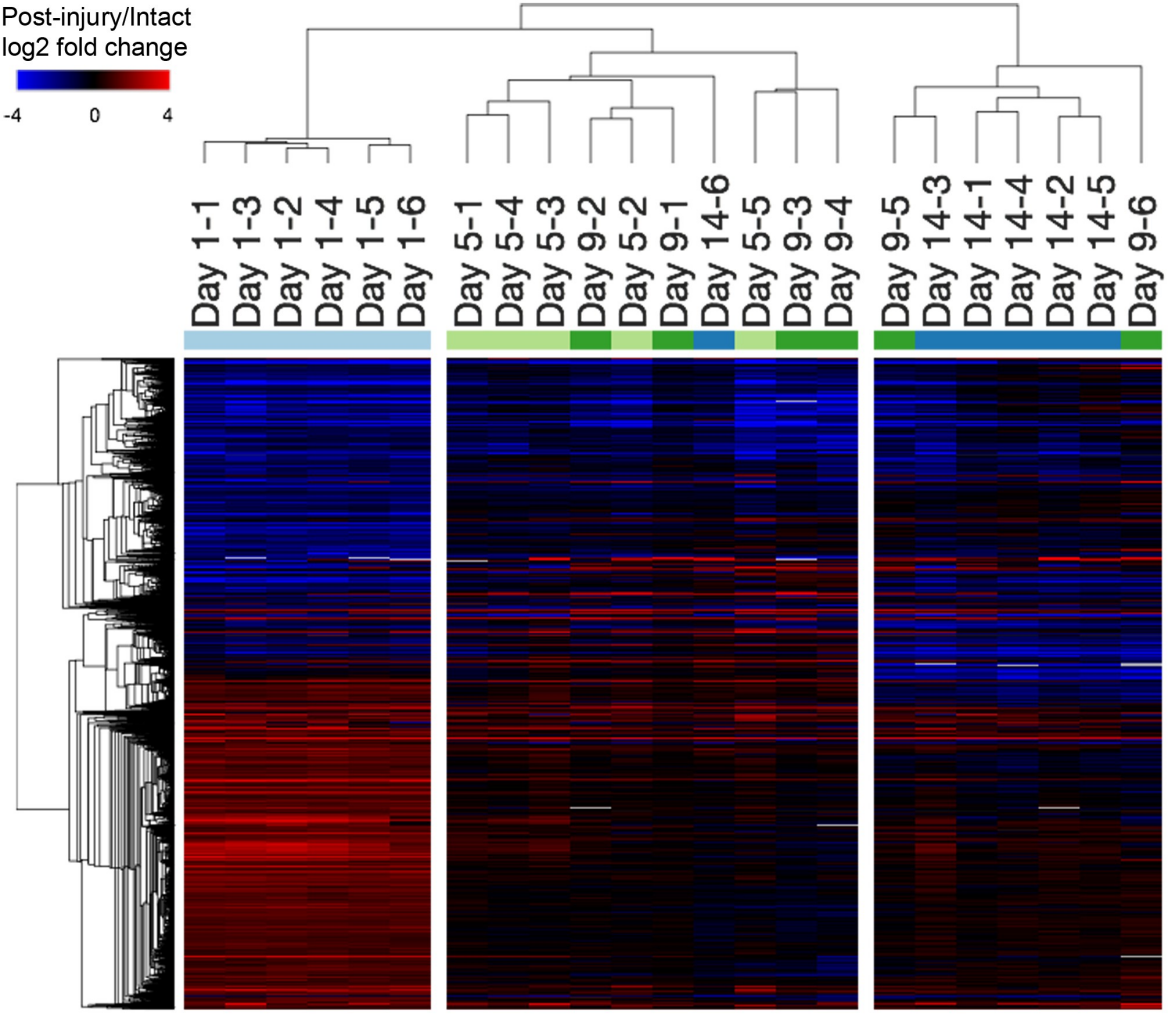
Transcriptional response of synovium to joint injury. Heatmap depicting the relative transcript abundance levels compared to intact controls (log-2 fold change of the individual post-injury specimens over the mean of the intact controls; red indicates higher expression following surgery, blue indicates lower expression). All 4109 transcripts (rows) differentially expressed in at least one of the assessed post-surgery time-points were included. Hierarchical clustering of post-surgery samples revealed 3 clusters: One distinct cluster containing all 1 day post-surgery samples, one cluster containing mostly 5 and 9 day post-surgery samples, and another containing mostly 14 day post-surgery samples, suggesting that the early transcriptional response at 1 day post-surgery is extensive and somewhat distinct from the later post-surgery time-points.

4109 transcripts were differentially expressed in at least one post-injury time-point compared to intact controls (Fig 1). Hierarchical clustering of post-injury samples revealed 3 clusters with similar gene expression profiles: one distinct cluster containing all 1-day post-injury samples, one cluster containing mostly 5- and 9-day post-injury samples, and another containing mostly 14-day post-injury samples (Fig 1). 3648 transcripts were differentially expressed at 1-day post-surgery compared to intact controls. 728, 615 and 813 transcripts were differentially expressed at 5, 9, and 14 days post-injury compared to intact controls, respectively. The top 10 most significant changes (based on p-values) at each post-injury time-point compared to intact controls are displayed in Table 1 (see Additional file 1 for full lists). The most significant change was found in *ACAN* expression, which was upregulated by 52-, 91-, and 60-fold at 5, 9, and 14 days post-injury, respectively.

**Table 1:** Transcriptional response of synovium to joint injury.

Rank	Gene symbol	Description	Intact RPKM	ACLT/Intact log2 FC	ACLT/Intact p-value
Day 1					
1	*PADI4*	Protein-arginine deiminase type-4	0.0	7.7	2.77E-92
2	*ARG1*	Arginase-1	0.1	6.4	5.34E-77
3	*NPM3*	Nucleoplasmin-3	10.3	4.9	1.53E-67
4	*TGM6 orthologue*	Protein-glutamine gamma-glutamyltransferase 6	1.9	5.1	5.12E-65
5	*CA12*	Carbonic anhydrase 12	0.5	5.1	2.73E-63
6	*SDS*	L-serine dehydratase/L-threonine deaminase	5.0	4.7	7.20E-57
7	*MCHR1*	Melanin-concentrating hormone receptor 1	0.3	5.0	5.18E-56
8	*BFSP1 orthologue*	Filensin	0.5	4.5	3.91E-55
9	*AMCF-II*	Alveolar macrophage chemotactic factor 2	1.6	4.3	2.80E-54
10	SELE	E-selectin	1.6	4.2	4.92E-53
Day 5					
1	*ACAN*	Aggrecan core protein	0.3	5.7	1.78E-80
2	*SFRP2*	Secreted frizzled-related protein 2	67.9	5.0	3.96E-72
3	-	Uncharacterized protein	0.3	5.1	1.71E-55
4	*CA12*	Carbonic anhydrase 12	0.5	4.9	1.04E-52
5	*MYPN*	Myopalladin	8.0	-6.3	6.60E-45
6	*MCHR1*	Melanin-concentrating hormone receptor 1	0.3	4.5	6.05E-44
7	*MMP1*	Interstitial collagenase	129.3	3.4	4.29E-36
8	*IGLV-3*	Immunglobulin	2.3	3.9	1.85E-34
9	*LCN2*	Neutrophil gelatinase-associated lipocalin	29.6	3.1	3.47E-34
10	-	Uncharacterized protein	16.5	-5.0	1.91E-33
Day 9					
1	*ACAN*	Aggrecan core protein	0.3	6.5	2.19E-106
2	*MMP9*	Matrix metalloproteinase-9	0.2	4.7	5.88E-64
3	*SFRP2*	Secreted frizzled-related protein 2	67.9	4.5	8.84E-64
4	*IDO1*	Indoleamine 2,3-dioxygenase 1	0.1	5.1	5.63E-58
5	*MYPN*	Myopalladin	8.0	-5.3	1.52E-43
6	*CRABP2*	Cellular retinoic acid-binding protein 2	10.9	3.3	7.05E-38
7	*ADAMTS16*	A disintegrin and metalloproteinase with thrombospondin motifs 16	3.4	3.4	2.64E-37
8	*COMP*	Cartilage oligomeric matrix protein	22.4	3.2	3.80E-36
9	-	Uncharacterized protein	33.5	3.3	1.09E-34
10	*FAIM2*	Protein lifeguard 2	5.8	-4.5	1.22E-34
Day 14					
1	*ACAN*	Aggrecan core protein	0.3	5.9	2.84E-89
2	*MYOC*	Myocilin	19.1	-9.0	4.71E-73
3	*MAP1LC3C*	Microtubule-associated proteins 1A/1B light chain 3C	9.0	4.8	7.27E-65
4	*IGLV-3*	Immunglobulin	2.3	4.4	9.72E-50
5	*VIT*	Vitrin	26.6	-5.1	3.46E-44
6	*IGKC*	Ig kappa chain C region	7.0	3.8	1.42E-42
7	*CA12*	Carbonic anhydrase 12	0.5	4.1	9.01E-41
8	*MMP1*	Interstitial collagenase	129.3	3.5	2.85E-40
9	*IGKJ2*	Uncharacterized protein	42.9	3.8	4.51E-40
10	*IGKJ3*	Uncharacterized protein	44.9	3.8	4.84E-40

The top 10 most significantly changed genes in comparison to intact controls are shown (see S1 Table for full lists). * RPKM = reads per kilobase of transcript per million mapped reads, ** orthologue

### Process network enrichment in synovium following joint injury

Twenty-three distinct process networks were significantly enriched on at least one of the post-injury time-points. Fifteen process networks were significantly enriched on the 1-day post-injury time-point, while 8, 8, and 7 process networks were significantly enriched on the 5, 9, and 14 days post-injury time-points, respectively (Table 2). Fourteen out of the 15 process networks significantly enriched on the 1-day post-injury time-point were unique to this time-point. Those predominantly included process networks related to cell cycling (i.e. *Cell cycle_Core*, *Cell cycle_G1-S*, *Cell cycle_G2-M*, *Cell cycle_Mitosis*, *Cell cycle_S phase*). At 5, 9, and 14 days after injury, there were 6 shared process networks amongst the respective 8, 8, and 7 significantly enriched process networks. Those were related to proteolysis (*Proteolysis_Connective tissue degradation*, *Proteolysis_ECM remodeling*), development (*Development_Cartilage development*, *Development_Ossification and bone remodeling*) and cell adhesion (*Cell adhesion_Cell-matrix adhesion*, *Cell adhesion_Platelet-endothelium-leucocyte interactions*).

**Table 2:** Process network enrichment in synovium following joint injury.

Process networks	Day 1 p-value	Day 5 p-value	Day 9 p-value	Day 14 p-value
Cell cycle				
*Cell cycle_Core*	2.11E-17	-	-	-
*Cell cycle_G1-S*	3.35E-05	-	-	-
*Cell cycle_G2-M*	1.17E-09	-	-	-
*Cell cycle_Mitosis*	2.18E-08	-	-	-
*Cell cycle_S phase*	1.05E-11	-	-	-
Others				
*Cytoskeleton_Spindle microtubules*	1.60E-12	-	-	-
*DNA damage_BER-NER repair*	2.56E-03	-	-	-
*DNA damage_Checkpoint*	4.06E-04	-	-	-
*Immune response_Phagosome in antigen presentation*	1.71E-03	-	-	-
*Protein folding_Folding in normal condition*	4.68E-04	-	-	-
*Proteolysis_Proteolysis in cell cycle and apoptosis*	4.55E-03	-	-	-
*Proteolysis_Ubiquitin-proteasomal proteolysis*	2.81E-06	-	-	-
*Transcription_mRNA processing*	2.49E-11	-	-	-
*Translation_Translation in mitochondria*	6.92E-07	-	-	-
*Inflammation_Complement system*	3.70E-06	2.26E-05	3.73E-06	-
Proteolysis				
*Proteolysis_ECM remodeling*	-	9.58E-07	2.13E-10	4.01E-06
*Proteolysis_Connective tissue degradation*	-	4.29E-07	1.00E-10	1.74E-07
Development				
*Development_Cartilage development*	-	5.24E-07	8.46E-11	1.76E-05
*Development_Ossification and bone remodeling*	-	8.87E-07	1.37E-04	5.45E-04
*Development_Regulation of angiogenesis*	-	3.17E-04	-	-
Cell adhesion				
*Cell adhesion_Cell-matrix interactions*	-	3.27E-17	2.09E-14	2.98E-06
*Cell adhesion_Platelet-endothelium-leucocyte interactions*	-	2.75E-04	6.76E-06	8.85E-04
*Cell adhesion_Integrin-mediated cell-matrix adhesion*	-	-	1.34E-03	1.69E-03

All process networks significantly enriched on the 1, 5, 9, or 14 days post-injury time-points were included.

### Proteolysis related gene expression changes

**Fig 2:**
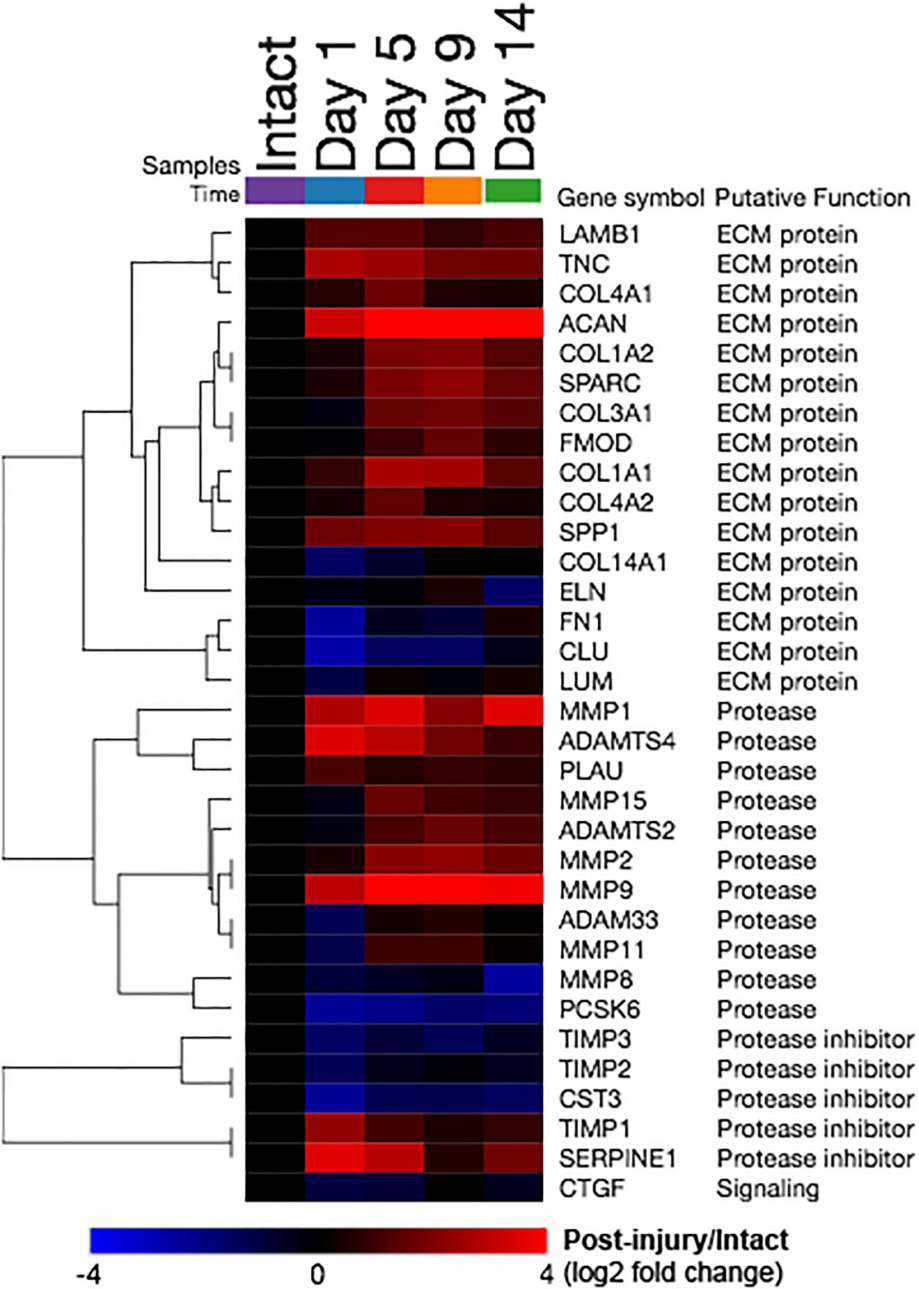
Proteolysis related gene expression changes (*Proteolysis_ECM remodeling* process network). Heatmap depicting the relative transcript abundance levels compared to intact controls (log-2 fold change from the comparison of post-injury group means over the mean of the intact controls). Red indicates higher expression following injury, blue indicates lower expression, i.e. significant increases of *MMP1* at 1, 5, 9, and 14 days post-injury were observed. All 33 transcripts (rows) differentially expressed in at least one of the assessed post-injury time-points were included (out of 83 total transcripts included in the process network).

Protease-encoding transcripts *MMP1*, *MMP2*, *MMP9*, *MMP15*, *ADAMTS2*, *ADAMTS4*, and *PLAU*, were more abundant in the synovium after ACL transection, while *ADAM33*, *MMP8* and *PCSK6* were significantly reduced after ACL transection. Protease inhibitor encoding *TIMP1* and *SERPINE1* were increased, while *TIMP3*, *TIMP2* and *CST3* were decreased. In addition, a group of ECM protein-coding transcript, including *COL1A1*, *COL1A2*, *COL3A1*, *COL4A1*, *COL4A2*, *ACAN*, *LAMB1*, *TNC*, *SPARC*, *FMOD* and *SPP1* were significantly increased, while expression of *COL14A1*, *ELN*, *FN1*, *CLU* and *LUM* were all significantly reduced following ACL transection. Out of 83 transcripts (corresponding to 72 total “network objects”) included in the *Proteolysis_ECM remodeling* process network, 33 transcripts were differentially expressed in at least one of the post-injury time-points compared to intact controls (Fig 2). In the synovial membrane of the intact knee, the most abundant protease-related transcripts were *TIMP2* and *TIMP3*; however, after surgical PTOA induction by the means of ACL transection, *MMP2* and *MMP1* became the most abundant. While the abundances of aggrecanase-associated transcripts peaked at 1, 5, or 9 days following surgery, the abundances of collagenase-associated transcripts peaked at 9 and 14 days after ACL transection.

### Cartilage development and ossification related gene expression changes

**Fig 3:**
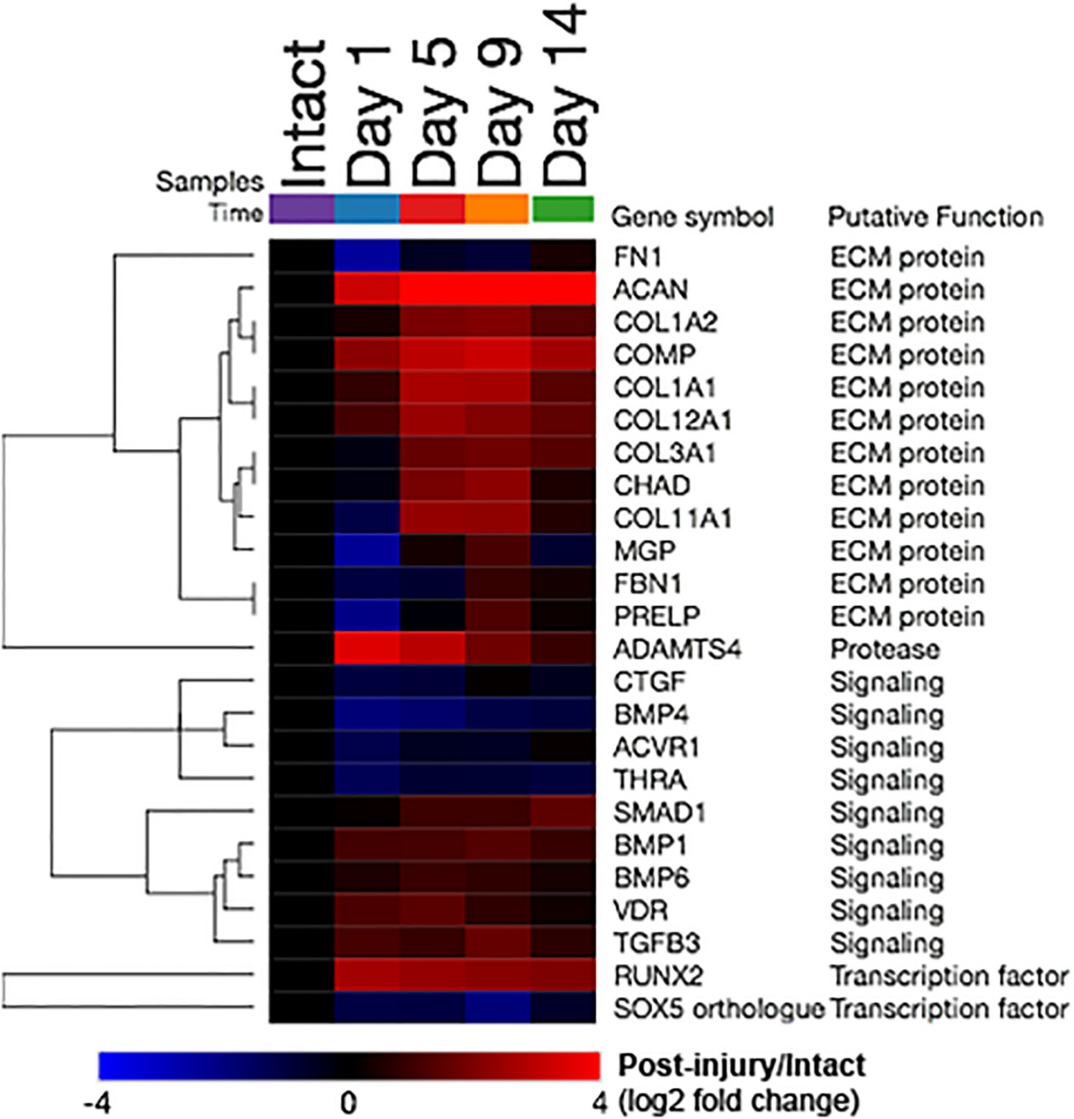
Cartilage development related gene expression changes (Development_Cartilage development process network). Heatmap depicting the relative transcript abundance levels compared to intact controls (log-2 fold change from the comparison of post-injury group means over the mean of the intact controls). Red indicates higher expression following injury, blue indicates lower expression, i.e. significant increases of *ACAN* at 1, 5, 9, and 14 days post-injury were observed. All 24 transcripts (rows) that are differentially expressed in at least one of the assessed post-injury time-points were included (out of 60 total transcripts included in the process network).

Out of 60 transcripts (corresponding to 61 total “network objects”) included in the *Development_Cartilage development* process network, 24 transcripts were differentially expressed in at least one of the post-injury time-points compared to intact controls (Fig 3). Among these transcripts, ECM protein-coding genes having increased expression included *ACAN*, *COL1A2*, *COMP*, *COL1A1*, *COL12A1*, *COL3A1*, *CHAD* and *COL11A1*. Signaling molecule-coding genes with increased expression included *SMAD1*, *BMP1*, *BMP6*, *VDR*, and *TGFB3*, while *CTGF*, *BMP4*, *ACVR1* and *THRA* were decreased. Among transcription factor-coding genes, *RUNX2* transcripts were increased from 1–14 days post-injury while *SOX5* orthologue transcripts were decreased.

### Cell adhesion related gene expression changes

Out of 168 transcripts (corresponding to 183 total “network objects”) included in the *Cell adhesion_Cell-matrix interactions* process network, 80 transcripts were differentially expressed in at least one of the post-injury time-points compared to intact controls (see S1 Fig). Most of the transcripts that contributed to the enrichment of the *Cell adhesion_Cell-matrix interactions* process network were ECM protein- and protease-coding transcripts that were described in the sections above. Amongst cell-membrane molecule-coding transcripts, a group with increased abundance included *GJA1*, *ITGA5*, *HMMR*, and a group with decreased abundance included *ITGAM*, *SGCG*, *LYVE1*, *SSPN*, *SGCE*, *SGCA*, *EGFR*, *ITGB5*, *ITGAX*, and *ITGB2*.

### Synovitis outcomes

**Fig 4:**
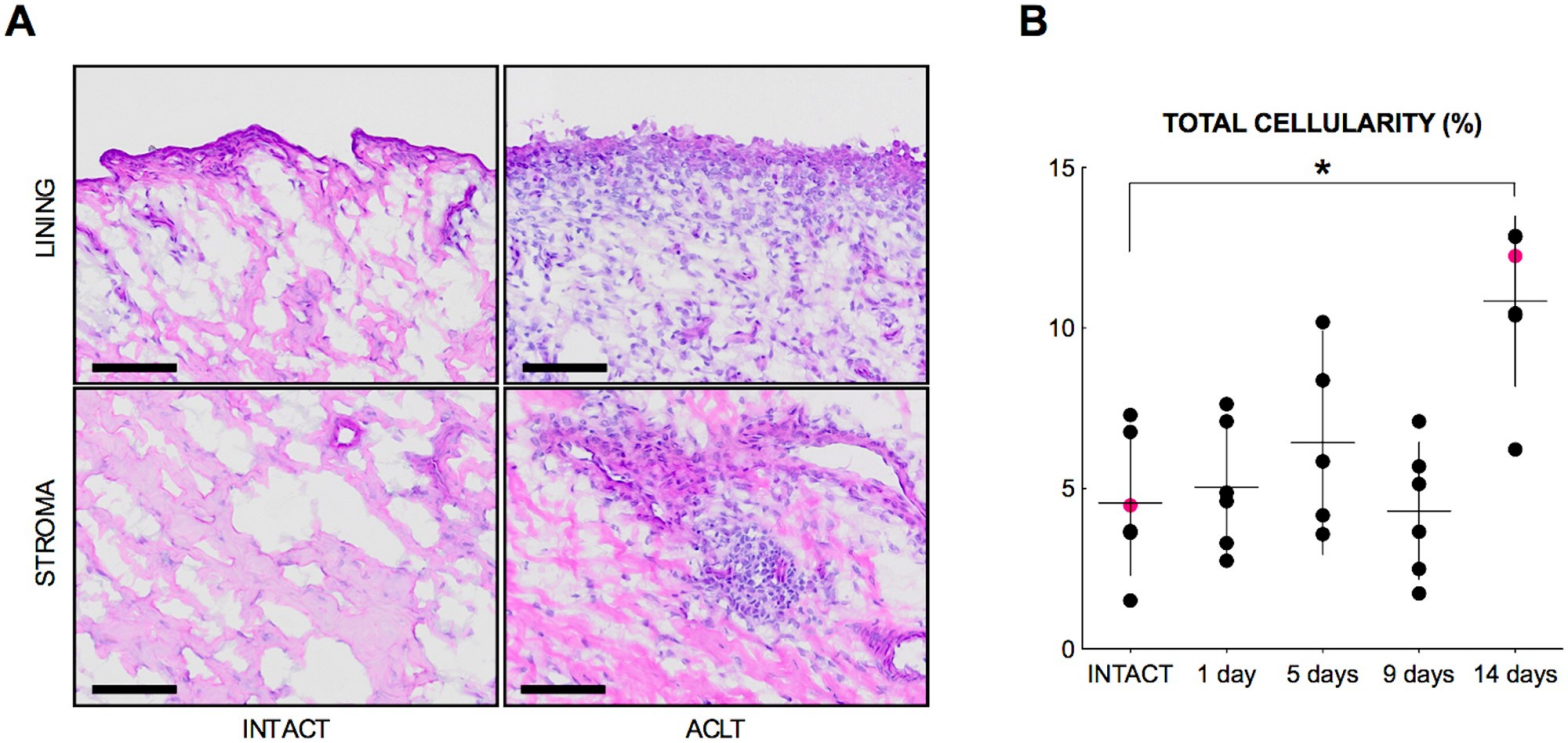
Histologic response of synovium to joint injury. (A) Photomicrographs of synovial biopsies obtained from intact controls and from knees at 14 days following injury, demonstrate an increased total cellularity at 14 days post-injury. Bars = 100 μm. (B) Symbols represent individual specimens, horizontal lines indicate the mean, and vertical bars indicate 95% confidence intervals. Magenta dots are corresponding to the selected photomicrographs, i.e. representing the median total cellularity of the respective time-points. * p < .05.

We detected a significant increase in total cellularity (whole biopsy) at 14 days post-injury (*p* = .036) when compared to the intact control group (Fig 4). The total cellularity measurements (whole biopsy) at 1, 5, and 9 days after injury were not different from intact controls (all p = 1.00). No substantial changes were observed in the microscopic sum score, lining score, stromal cellularity score and infiltration score (Table 3).

**Table 3:** Histopathological synovium outcomes.

Scale	Total cellularity (Whole biopsy)		Microscopic sum score		Lining score		Stromal cellularity score		Infiltration score	
	0–100%		0–9		0–3		0–3		0–3	
	*Mean (95% CI)*	*p*	*Median (Range)*	*p*	*Median (Range)*	*p*	*Median (Range)*	*p*	*Median (Range)*	*p*
**INTACT**	4.55 (2.83, 6.28)	-	4.5 (2–5)	-	1.0 (0–1)	-	2.0 (1–3)	-	1.0 (1–2)	-
**DAY 1**	5.04 (3.46, 6.62)	1	4.0 (3–5)	1	1.0 (1–2)	1	2.0 (1–2)	1	1.0 (1–2)	1
**DAY 5**	6.43 (3.97, 8.89)	1	4.0 (3–5)	1	0.0 (0–1)	1	2.0 (1–2)	1	2.0 (1–2)	1
**DAY 9**	4.30 (2.67, 5.93)	1	4.5 (2–6)	1	1.0 (0–2)	1	2.0 (1–2)	1	1.0 (1–2)	1
**DAY 14**	10.84 (8.82, 12.85)	0.036	5.5 (4–7)	1	1.5 (0–3)	1	2.0 (2–2)	1	2.0 (1–2)	0.21

## Discussion

The results of this study confirmed our hypotheses that significant enrichment of protease-related processes would be found in the synovium after ACL transection, with enrichment identified at days 5 to 14 after surgery. In addition, transcripts for *MMP1* and *ADAMTS4* (encoding proteases which degrade extracellular matrix proteins of cartilage and are found in the synovial fluid of injured and arthritis knees) were increased in the synovium in this same post-operative period. In the synovial membrane of the intact knee, the most abundant protease-related transcripts were *TIMP2* and *TIMP3*; however, after surgical PTOA induction by the means of ACL transection, *MMP2* and *MMP1* became the most abundant. RNA-seq also identified transcriptional enrichment for cell proliferation at day 1, as well as enrichment of cartilage development and cell adhesion networks at 5 to 14 days following ACL transection. A functional consequence of the early upregulation of cellular proliferation networks was reflected by the measured increase in cellularity in the synovium at 14 days after ACL transection (*p* = 0.003). In addition, the increased expression of genes encoding for *MMP1* and *ADAMTS4* was consistent with prior reports of the presence of these proteases in the synovial fluid of injured and arthritic joints [13–15].

The transcriptional changes in the synovium at 1 day post-injury was distinct from that observed at day 5, 9 and 14 days post-injury, in terms of the number of differentially expressed genes (i.e., 3648, 728, 615, and 813 at 1, 5, 9, and 14 days post-injury, respectively), and the number of significantly enriched process networks (i.e., 15, 8, 8, and 7, respectively). While 14 process networks were unique to day 1, there was a large overlap between the enriched networks at 5, 9, and 14 days post-injury, suggesting similar transcriptional activity in the synovium at these time-points. Taking all transcriptome-wide changes into account, the major processes elicited in the synovium following knee injury were related to cell-cycling (unique to 1-day post-injury), as well as proteolysis, cell-adhesion, and development, specifically cartilage development (shared at 5, 9, and 14 days post-surgery).

Proteolysis-related transcripts that increased in synovium included *MMP1*, *MMP2*, *MMP9*, *MMP15*, *ADAMTS2*, *ADAMTS4*, and *PLAU*; few protease-encoding transcripts decreased. Therefore, our data suggest a significant proteolytic response in the synovium to ACL injury at the transcriptional level, consistent with previously available human[7–10, 12] and minipig data[19, 25]. The temporal expression profiles provided here agree with previously highlighted roles of some proteases, i.e. those encoded by *MMP1*, *MMP2*, and *ADAMTS4*, while additional molecules identified here might be considered as novel targets for therapeutic approaches, such as *MMP9*, *MMP15*, *ADAMTS2* and *PLAU*.

Interestingly, along with the proteases, there was increased expression of ECM proteins, suggesting both proteolysis and matrix synthesis were occurring in the synovium. The ~ 60-fold increased expression of *ACAN*, encoding cartilage ECM proteoglycan aggrecan was the most significant change detected in the synovium at 5, 9, and 14 days post-injury. The change in synovial *ACAN* expression was accompanied by the increased expression of other genes encoding cartilage ECM components such as *COMP*, *SPARC* and *TNC*, altogether suggesting increased expression of cartilage ECM proteins by the synovial cells. This is consistent with prior reports of increased abundance of transcripts for ECM molecules including *ACAN*, *COMP*, *SPARC* and *TNC*, at 4 weeks post PTOA induction[19]. However, it is currently unclear whether the activation of cartilage development and ossification-related transcripts in the synovium following injury is a protective or pathogenic mechanism, or a marker of PTOA development.

Cell-adhesion related transcriptional changes in the synovium included those associated with cell matrix interactions, as well as those associated with platelet-endothelium-leucocyte interactions. Amongst the increased cell-surface molecule-encoding transcripts were *ITGA5* and *GJA1*. *ITGA5* encodes the integrin subunit alpha 5, which can assemble with the integrin subunit beta 1 to form Integrin alpha-5/beta-1, a receptor for fibronectin and fibrinogen. *GJA1* encodes gap junction protein alpha 1, which is a connexin family member, and as such a component of gap junctions. Both, *ITGA5* and *GJA1* have a role in the development of synovial joints as well [35,36]. *ITGA5* is downregulated in the interzone, the site that gives rise to synovium, articular cartilage and other intraarticular structures, and its downregulation is required to allow for normal joint formation [40, 41]. Genetically modified mice in which *Itga5* was deleted throughout interzone derived cells using a *Gdf5*-Cre driver, were protected from the progression of cartilage damage following medial collateral ligament transection and meniscectomy[42]. In contrast to *ITGA5*, *GJA1* is predominantly expressed in the interzone, but its role in joint development and PTOA is not yet defined. Taken together, since *Itga5* deficient mice were protected from PTOA following joint injury and we observe increased *ITGA5* expression post-injury, the inhibition of the integrin alpha-5/beta-1 binding site might be an effective therapeutic strategy for PTOA. In addition to the aforementioned changes in cells with mesenchymal origin, platelet-endothelium-leucocyte interactions might be initiated following joint injury. In order to assess whether an increased extravasation of leukocytes into the synovium is initiated post-injury, we assessed the cellularity throughout the synovial biopsies. While the cellularity remained relatively unchanged throughout 1 to 9 days post-surgery, we detected a significant increase in synovial cellularity at 14 days post-injury. Increased cellularity is a histological feature of synovitis. It is unclear whether the observed increase in cellularity was due to the cell-adhesion-related changes enriched at 5–9 days post-injury, or due to the cell-cycling, or specifically mitosis-related changes observed at 1-day post-injury.

Our study has a few limitations. One was the lack of a sham control group, where only the arthrotomy was made without the ACL injury so that the effect of synovial injury alone could be assessed. However, when modeling the human condition, it is well-established that at the time of ACL rupture, the synovium is also damaged in most patients [22, 23]. Thus, while the use of a sham control with the synovial injury due to the incision would be of scientific interest, a model where both synovium and ACL injury occur is more representative of the human condition that promotes osteoarthritis. Furthermore, to minimize the local effects of synovial damage in our model, we systematically sampled the synovium at locations far from the synovium damaged at the time of surgery. Another limitation is that even though we obtained >10 million paired-end reads/library, this amount of sequencing only provided statistical power to detect changes in mRNA abundance >1.6-fold for transcripts having RPKM values >5. Consequently, genes encoding inflammatory components such as IL1β or IL6 may have been expressed at a lower level and hence we would be unable to detect them. Future studies using alternative techniques, including immunohistochemistry or quantitative PCR, may provide additional insight in the upregulation of pro-inflammatory genes that may also play a role in the post-traumatic knee. In addition, the porcine ACL transection model was used to control injury type and time post-injury, and to permit tissue harvests and may not be fully representative of the human condition. Nonetheless, the pattern of PTOA development between the pig and the human has been shown to be similar [5]. Finally, the long-term implications of the acute findings are not fully known.

## Conclusions

The synovial membrane is an intra-articular tissue, with a broad surface area and abundant vascularization, making it a logical target for disease modifying therapies. We observed a multitude of synovial gene expression changes with transcriptome-wide significance, in the early response to a surgical knee injury known to reliably cause PTOA. These changes resulted in enrichment of processes related to cell cycle (unique to 1-day post-injury), proteolysis, cartilage development and cell adhesion (shared at 5, 9, and 14 days post-injury) and preceded histopathological signs of synovitis. Increased expression of transcripts associated with catabolic processes (proteolysis) and anabolic processes (cartilage development) suggests that the synovium may play a complex, balancing role in the early response to PTOA induction, and that the synovium should be included in future studies of the synovial joint response to injury and the subsequent development of osteoarthritis.

## Supporting information

S1 Fig Cell adhesion related gene expression changes (Cell adhesion_Cell-matrix interactions). Heatmap depicting the relative transcript abundance levels compared to intact controls (log-2 fold change from the comparison of post-injury group means over the mean of the intact controls). Red indicates higher expression following injury, blue indicates lower expression.
(TIFF)

S1 Table Transcriptional response of synovium to joint injury. All significant transcriptome-wide gene expression changes are included.
(XLSX)

S1 Checklist The ARRIVE guidelines checklist. (DOCX)
