# Supplementary figures and images for: Proteolysis and cartilage development are activated in the synovium after surgical induction of post traumatic osteoarthritis
Source: PLoS One. 2020 Feb 27;15(2):e0229449. doi: 10.1371/journal.pone.0229449 (PMC7046188; doi:10.1371/journal.pone.0229449)

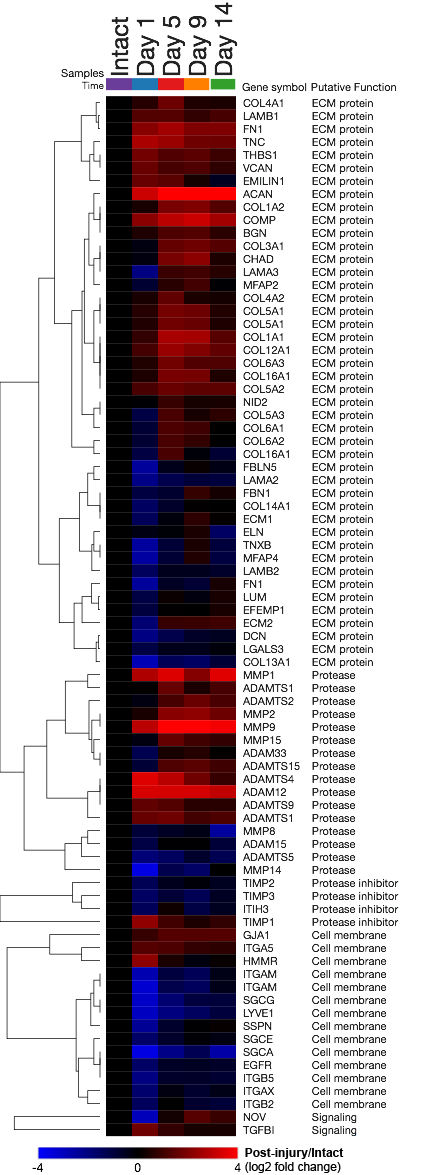

Supplement: S1 Fig — Heatmap depicting the relative transcript abundance levels compared to intact controls (log-2 fold change from the comparison of post-injury group means over the mean of the intact controls). Red indicates higher expression following injury, blue indicates lower expression. (TIFF) [file pone.0229449.s001.tiff]
