# Supplementary material for: Proteolysis and cartilage development are activated in the synovium after surgical induction of post traumatic osteoarthritis
Source: PLoS One. 2020 Feb 27;15(2):e0229449. doi: 10.1371/journal.pone.0229449 (PMC7046188; doi:10.1371/journal.pone.0229449)

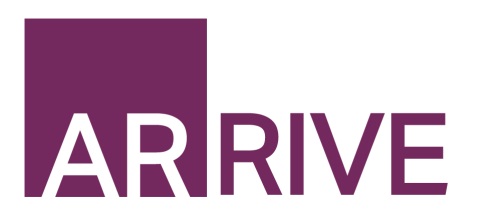


The ARRIVE Guidelines Checklist

Animal Research: Reporting In Vivo Experiments

Carol Kilkenny^1^, William J Browne^2^, Innes C Cuthill^3^, Michael Emerson^4^ and Douglas G Altman^5^

*^1^The National Centre for the Replacement, Refinement and Reduction of Animals in Research, London, UK, ^2^School of Veterinary Science, University of Bristol, Bristol, UK, ^3^School of Biological Sciences, University of Bristol, Bristol, UK, ^4^National Heart and Lung Institute, Imperial College London, UK, ^5^Centre for Statistics in Medicine, University of Oxford, Oxford, UK.*

|  | | ITEM | RECOMMENDATION | Section/ Paragraph |
| --- | --- | --- | --- | --- |
| 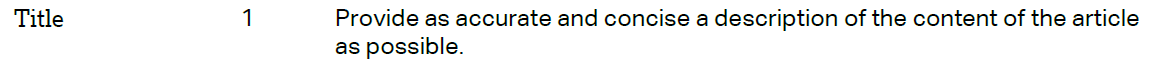 | | | Title Page |  |
| 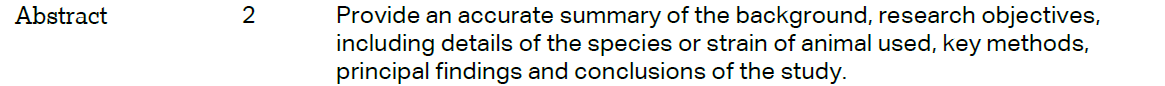 | | | Abstract |  |
| INTRODUCTION | | |  |  |
| 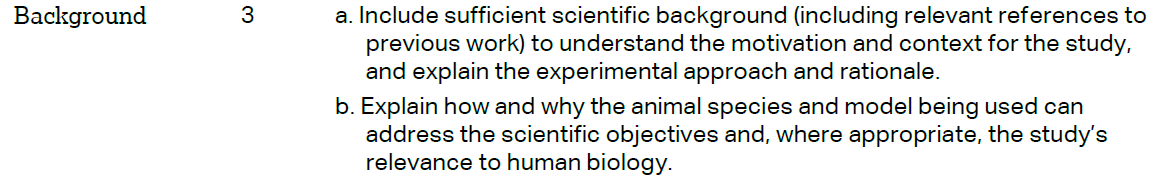 | | | Introduction  Introduction, last paragraph |  |
| 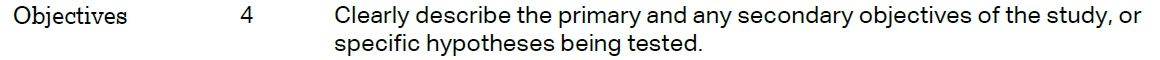 | | | Page 6 |  |
| METHODS | | |  |  |
| 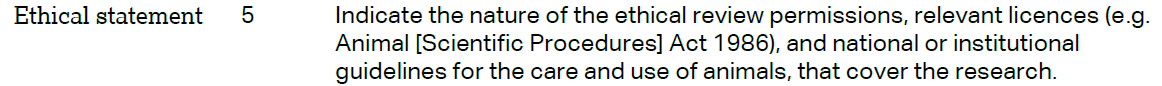 | | | Methods, first paragraph |  |
| 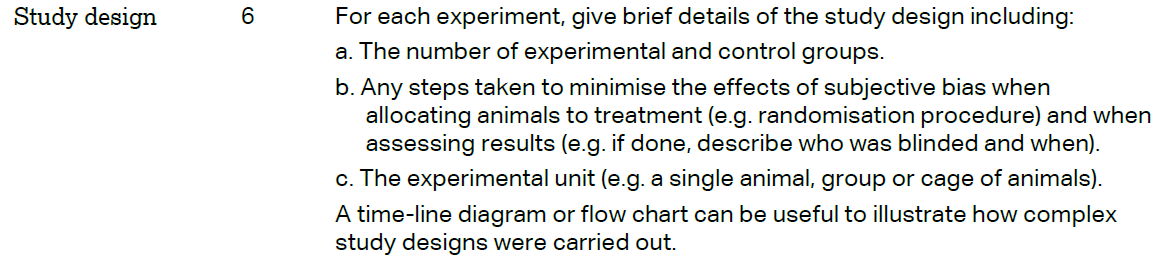 | | | Methods, first paragraph |  |
| 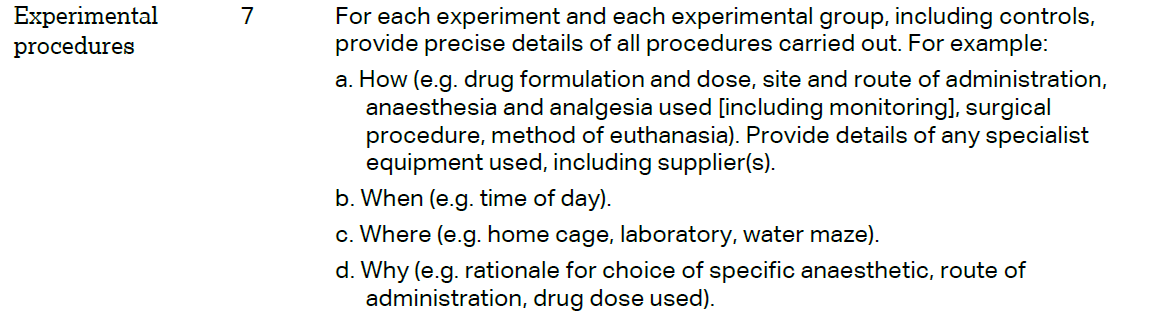 | | | Methods, first paragraph. |  |
| 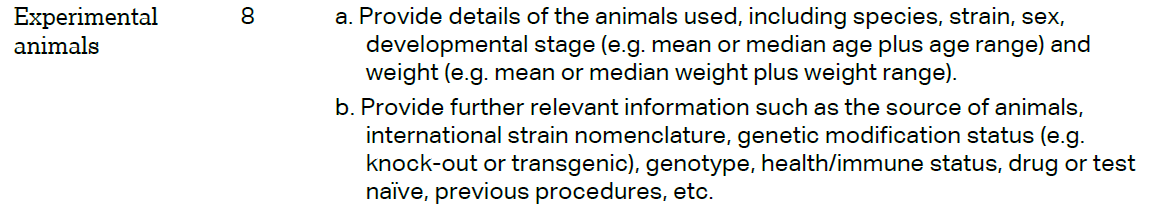 | | | Methods, first paragraph. |  |

The ARRIVE guidelines. Originally published in *PLoS Biology*, June 2010^1^

| 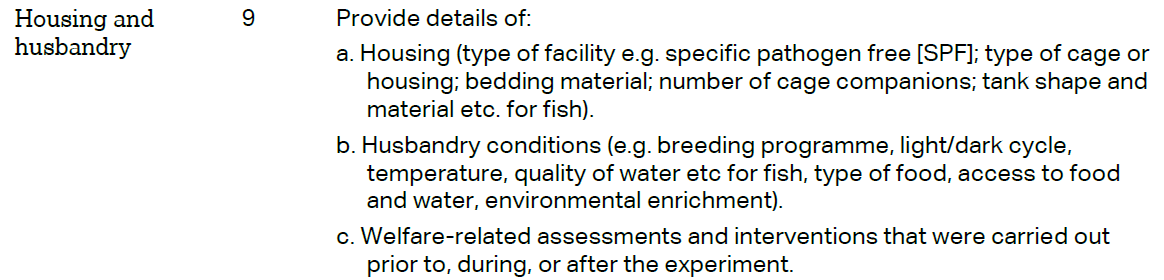 | Methods, first paragraph | |
| --- | --- | --- |
| 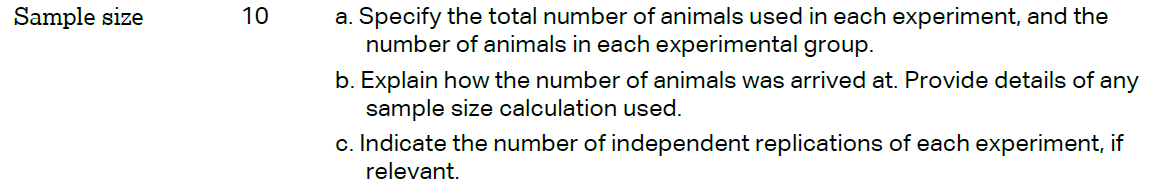 | Methods, first paragraph | |
| 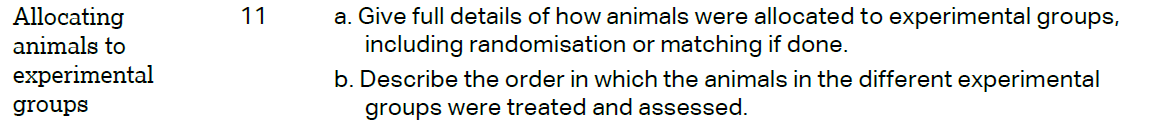 | Methods, first paragraph | |
| 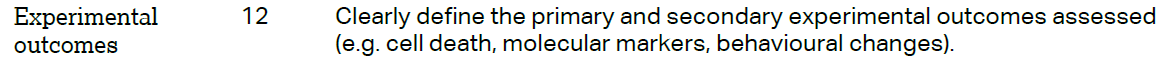 | Methods, pages 9 and 10 | |
| 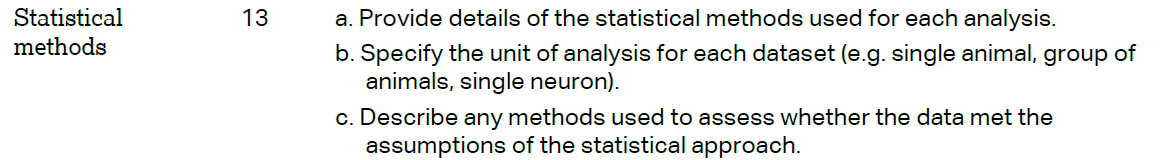 | Methods, RNA-Seq data analysis, last paragraph | |
| RESULTS |  | |
| 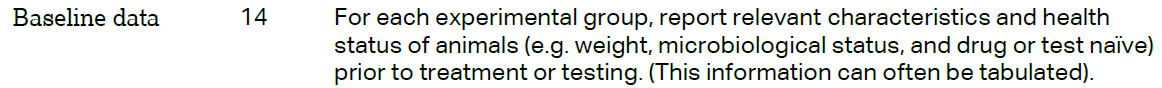 | Methods, first paragraph | |
| 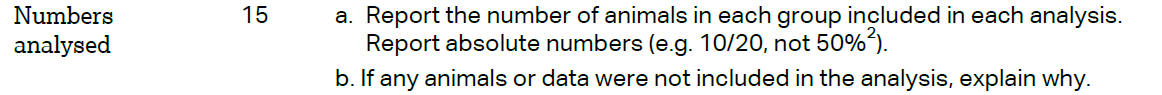 | Methods, first paragraph | |
| 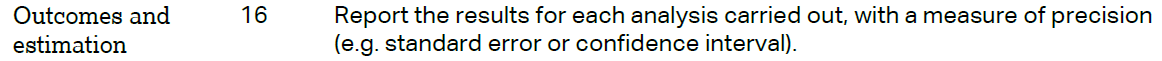 | Results | |
| 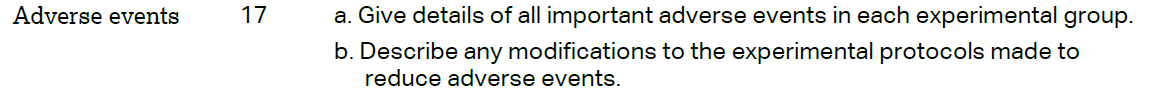 | No important adverse events | |
| DISCUSSION |  | |
| 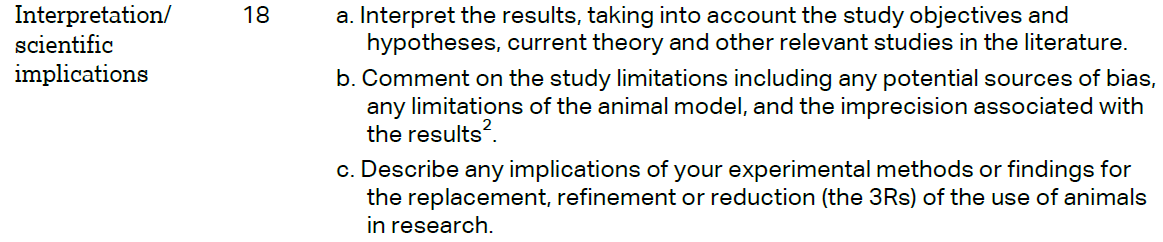 | Discussion | |
| 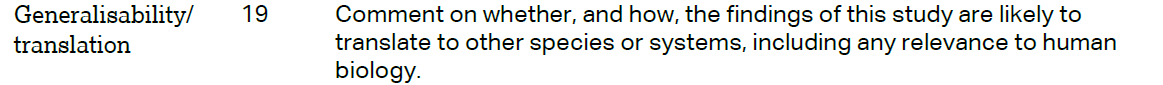 | Conclusion | |
| 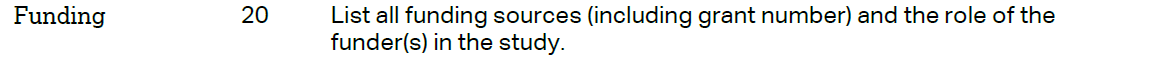 | | Acknowledgements |


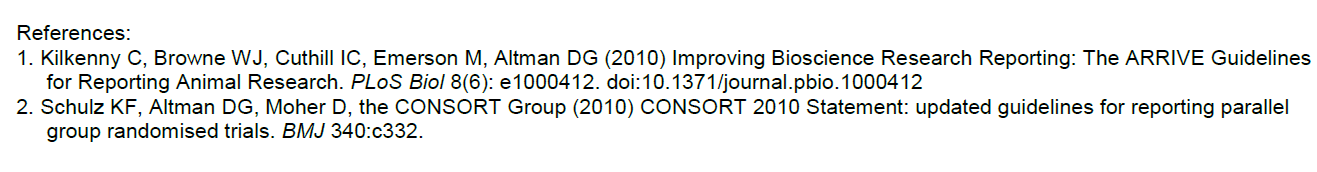

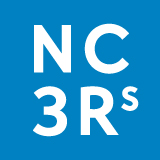

Supplement: S1 Checklist — (DOCX) [file pone.0229449.s003.docx]
